# Supplementary material for: Technological Changes in Wheat-Based Breads Enriched with Hemp Seed Press Cakes and Hemp Seed Grit
Source: Molecules. 2022 Mar 11;27(6):1840. doi: 10.3390/molecules27061840 (PMC8954276; doi:10.3390/molecules27061840)
Supplement: Supplementary file 1 [file molecules-27-01840-s001.zip › molecules-1596207-SI.pdf]

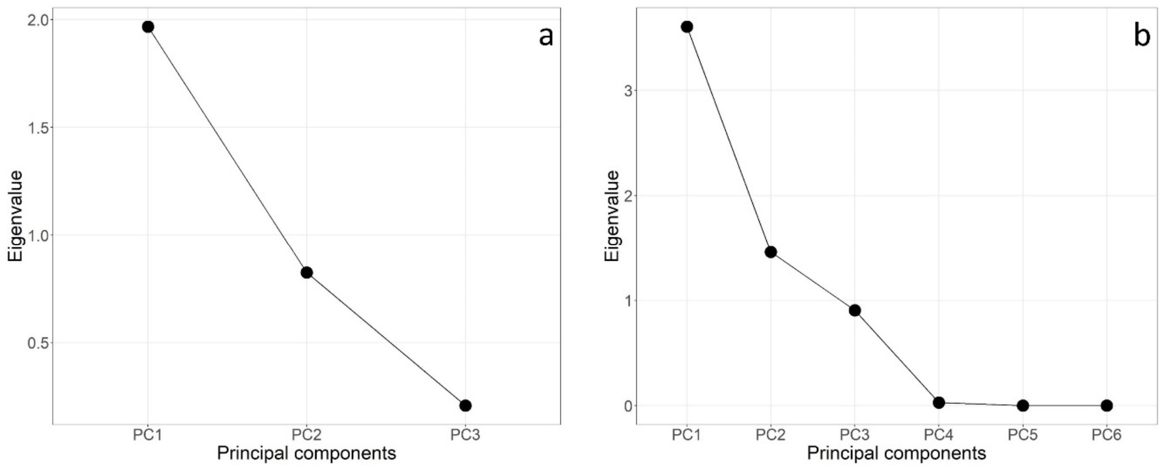


**Supplementary Figure S1.** Screeplots of scaled and centeres principal component analysis of dough and bread colours (**a**) and dough and bread textures (**b**).

**Supplementary Table S1.** Medians water binding capacity (WBC) and least gelation concentration (LGC) of raw materials.

| Sample | WBC / % | LGC / % |
|---|---|---|
| HSG | 2.0 | 16 |
| HPF | 1.4 | 18 |
| HP46 | 1.5 | 14 |
| HP54 | 1.2 | 22 |
| WFM | 1.1 | 16 |

**Supplementary Table S2.** Median colour measurements of raw materials, doughs and breads.

| Sample | L* | a* | b* |
|---|---|---|---|
| HSG | 55.7 | 3.1 | 12.6 |
| HPF | 60.1 | 5.2 | 17.9 |
| HP46 | 55.2 | 5.4 | 18.7 |
| HP54 | 73.0 | 9.4 | 25.2 |
| WFM | 89.2 | 3.4 | 10.0 |
| HSG-1-D | 71.6 | 8.0 | 18.3 |
| HPF-1-D | 68.8 | 7.8 | 19.4 |
| HP46-1-D | 69.2 | 7.9 | 19.3 |
| HP54-1-D | 70.8 | 8.6 | 19.5 |
| HPF-18-D | 51.4 | 5.4 | 16.9 |
| HP46-14-D | 54.1 | 5.6 | 19.1 |
| HP54-10-D | 67.2 | 9.3 | 23.8 |
| WFM-D | 71.5 | 8.6 | 19.0 |
| HSG-1-B | 64.9 | 8.0 | 17.3 |
| HPF-1-B | 59.4 | 8.3 | 17.8 |
| HP46-1-B | 59.8 | 7.8 | 17.4 |
| HP54-1-B | 63.0 | 9.1 | 19.4 |

| | | | |
|---|---|---|---|
| HPF-18-B | 43.5 | 4.2 | 8.3 |
| HP46-14-B | 47.8 | 5.2 | 11.8 |
| HP54-10-B | 56.9 | 9.7 | 20.5 |
| WFM-B | 64.0 | 8.6 | 18.1 |

**Supplementary Table S3.** Medians for stickiness of dough (D) and hardness, cohesion, elasticity, gumminess, chewiness of breads (B).

| | Stickiness (D) / N | Hardness (B) / N | Cohesion (B) | Elasticity (B) | Gumminess (B) / N | Chewiness (B) / N |
|---|---|---|---|---|---|---|
| HSG-1 | -0.66 | 7.84 | 0.97 | 1.06 | 7.62 | 8.09 |
| HPF-1 | -0.35 | 8.58 | 0.92 | 1.06 | 8.09 | 8.60 |
| HP46-1 | -0.53 | 6.22 | 0.96 | 1.05 | 6.06 | 6.35 |
| HP54-1 | -0.25 | 13.61 | 0.88 | 1.06 | 12.05 | 12.82 |
| HPF-18 | -0.70 | 14.70 | 0.90 | 1.09 | 13.26 | 14.45 |
| HP46-14 | -0.70 | 15.55 | 0.92 | 1.10 | 14.04 | 15.30 |
| HP54-10 | -0.42 | 7.74 | 1.11 | 1.13 | 7.84 | 8.77 |
| WFM | -0.55 | 6.00 | 1.05 | 1.08 | 6.31 | 6.81 |

**Supplementary Table S4.** Loadings for principle component analysis of raw materials, doughs and breads.

| **Loading for PC1–5 of PCA of raw materials (figure 2)** | | | | | |
|---|---|---|---|---|---|
| **Parameter** | **PC1** | **PC2** | **PC3** | **PC4** | **PC5** |
| Dry matter | 0.18282 | 0.25964 | 0.47309 | 0.16624 | -0.49612 |
| Water binding capacity | -0.36067 | 0.23391 | 0.00062 | 0.03413 | 0.22485 |
| Least gelation concentration | 0.29910 | 0.18229 | 0.27958 | -0.73206 | -0.08884 |
| d(0.5) | -0.37552 | 0.16181 | 0.17965 | -0.22127 | 0.27481 |
| RR | 0.13863 | -0.41068 | 0.13523 | -0.14887 | -0.23983 |
| Energy | -0.35566 | 0.11197 | 0.334024 | -0.00595 | 0.05236 |
| Protein | 0.24813 | 0.34501 | 0.02958 | 0.42121 | 0.16972 |
| Fat | -0.35422 | 0.19631 | 0.21703 | -0.17020 | -0.06459 |
| Carbo-hydrates | -0.00833 | -0.43403 | 0.15543 | 0.08662 | 0.00214 |
| Dietary Fiber | 0.08137 | 0.18452 | -0.59916 | -0.37174 | -0.00155 |
| L* | 0.14746 | -0.37862 | 0.27280 | -0.10215 | 0.59325 |
| a* | 0.37142 | 0.18517 | 0.16855 | 0.01398 | 0.36955 |
| b* | 0.32692 | 0.28217 | 0.04397 | 0.03775 | 0.19532 |
| **Loading for PCA of doughs (figure 3a)** | | | | | |
| **Parameter** | **PC1** | **PC2** | **PC3** | **PC4** | **PC5** |
| L* | 0.39145 | 0.92006 | -0.01584 | | |
| a* | 0.65239 | -0.26534 | 0.70992 | | |
| b* | 0.64897 | -0.28823 | -0.70410 | | |
| **Loading for PC1–5 PCA of breads (figure 3b)** | | | | | |
| **Parameter** | **PC1** | **PC2** | **PC3** | **PC4** | **PC5** |
| Hardness | 0.52532 | 0.02613 | -0.05242 | 0.21382 | -0.80387 |
| Cohesion | -0.38481 | -0.55038 | -0.09878 | 0.73119 | -0.06313 |
| Elasticity | 0.13209 | -0.75596 | -0.32277 | -0.54789 | -0.07139 |
| Gumminess | 0.52360 | -0.05844 | -0.07011 | 0.27043 | 0.25652 |
| Chewiness | 0.52033 | -0.10839 | -0.07715 | 0.20590 | 0.52794 |
| Stickiness | 0.11671 | -0.33131 | 0.93405 | -0.06270 | -0.01360 |
